# Structural basis for the activity and specificity of the immune checkpoint inhibitor lirilumab

**DOI:** 10.1038/s41598-023-50262-6

**Published:** 2024-01-07

**Authors:** Nicholas Lorig-Roach, Nina M. Harpell, Rebecca M. DuBois

**Affiliations:** Department of Biomolecular Engineering, University of California Santa Cruz, Santa Cruz, CA USA

**Keywords:** X-ray crystallography, Cancer immunotherapy

## Abstract

The clinical success of immune checkpoint inhibitors has underscored the key role of the immune system in controlling cancer. Current FDA-approved immune checkpoint inhibitors target the regulatory receptor pathways of cytotoxic T-cells to enhance their anticancer responses. Despite an abundance of evidence that natural killer (NK) cells can also mediate potent anticancer activities, there are no FDA-approved inhibitors targeting NK cell specific checkpoint pathways. Lirilumab, the most clinically advanced NK cell checkpoint inhibitor, targets inhibitory killer immunoglobulin-like receptors (KIRs), however it has yet to conclusively demonstrate clinical efficacy. Here we describe the crystal structure of lirilumab in complex with the inhibitory KIR2DL3, revealing the precise epitope of lirilumab and the molecular mechanisms underlying KIR checkpoint blockade. Notably, the epitope includes several key amino acids that vary across the human population, and binding studies demonstrate the importance of these amino acids for lirilumab binding. These studies reveal how KIR variations in patients could influence the clinical efficacy of lirilumab and reveal general concepts for the development of immune checkpoint inhibitors targeting NK cells.

## Introduction

Despite decades of progress in the detection and treatment of cancer, its myriad of forms and variations persist as some of the biggest threats to human health^1^. While small molecule chemotherapeutic agents remain the frontline of non-surgical treatment, a number of monoclonal antibody biologics have become available for specialized indications, including antibodies targeting cancer cells themselves (e.g. rituximab targeting CD20 in leukemia and lymphoma; trastuzumab targeting HER2 for breast cancer) or targeting growth factors that promote tumor growth (e.g. bevacizumab targeting VEGF-A). Monoclonal antibodies’ precise and selective targeting of almost any molecular motif, their relative ease of production, and ability to pair with immune effector systems continue to make them strong therapeutic candidates.

In the past decade, a new class of monoclonal antibody biologic termed immune checkpoint inhibitors has emerged. Immune checkpoint inhibitors modulate regulatory receptors on cytotoxic T-cells and natural killer (NK) cells to improve clearance of cancer cells^2–6^. Cytotoxic T-cells and NK cells play a pivotal role in the immune system’s response to malignancy, using a variety of strategies to surveil for the presence of non-self with a mixture of activating and inhibitory receptors, often interacting with MHC molecules^7^. In the presence of strong activating receptor signals or a lack of inhibitory receptor signals, T-cells and NK cells are activated and secrete cytolytic molecules, cytokines, and chemokines that kill nearby cancerous cells and recruit additional immune effector cells^8^. Immune checkpoint inhibitors seek to tip the balance further toward activation by blocking inhibitory receptors such as CTLA-4, PD-1, LAG-3, and KIRs, and preventing tumors from escaping detection via inhibitory signaling^4^. The FDA has approved the use of several antibodies that target PD-1/PD-L1 (nivolumab, pembrolizumab, atezolizumab, and others), CTLA-4 (ipilimumab, tremelimumab), and LAG-3 (relatlimab) for a number of indications, including melanoma, head and neck cancer, lung cancer, renal cancer and liver cancer^9^.

Among immune checkpoint inhibitor targets, the killer immunoglobulin-like receptor (KIR) family is a candidate for the enhancement of NK cell mediated tumor clearance^10^. KIRs recognize the basally expressed MHC class I molecules, conferring NK cells the ability to detect self. The KIR family is composed of a number of both inhibitory and activating receptors which differ in the number of extracellular Ig-like domains they contain (KIR**2D**L1 vs KIR**3D**L1), the length of their cytoplasmic tail (KIR2DL1 vs KIR2DS1), and sequence variation (KIR2DL1 vs KIR2DL3). It has been known for some time that there is significant population level diversity in both KIR and MHC genes, and different KIR receptors and their variants pair with distinct HLA molecules^11,12^. The binding kinetics between KIRs and HLA are characterized by rapid on- and off-rates with affinities ranging from high nanomolar to micromolar depending on the variant pairing and the peptide presented by the HLA^13,14^.

**Figure 1:**
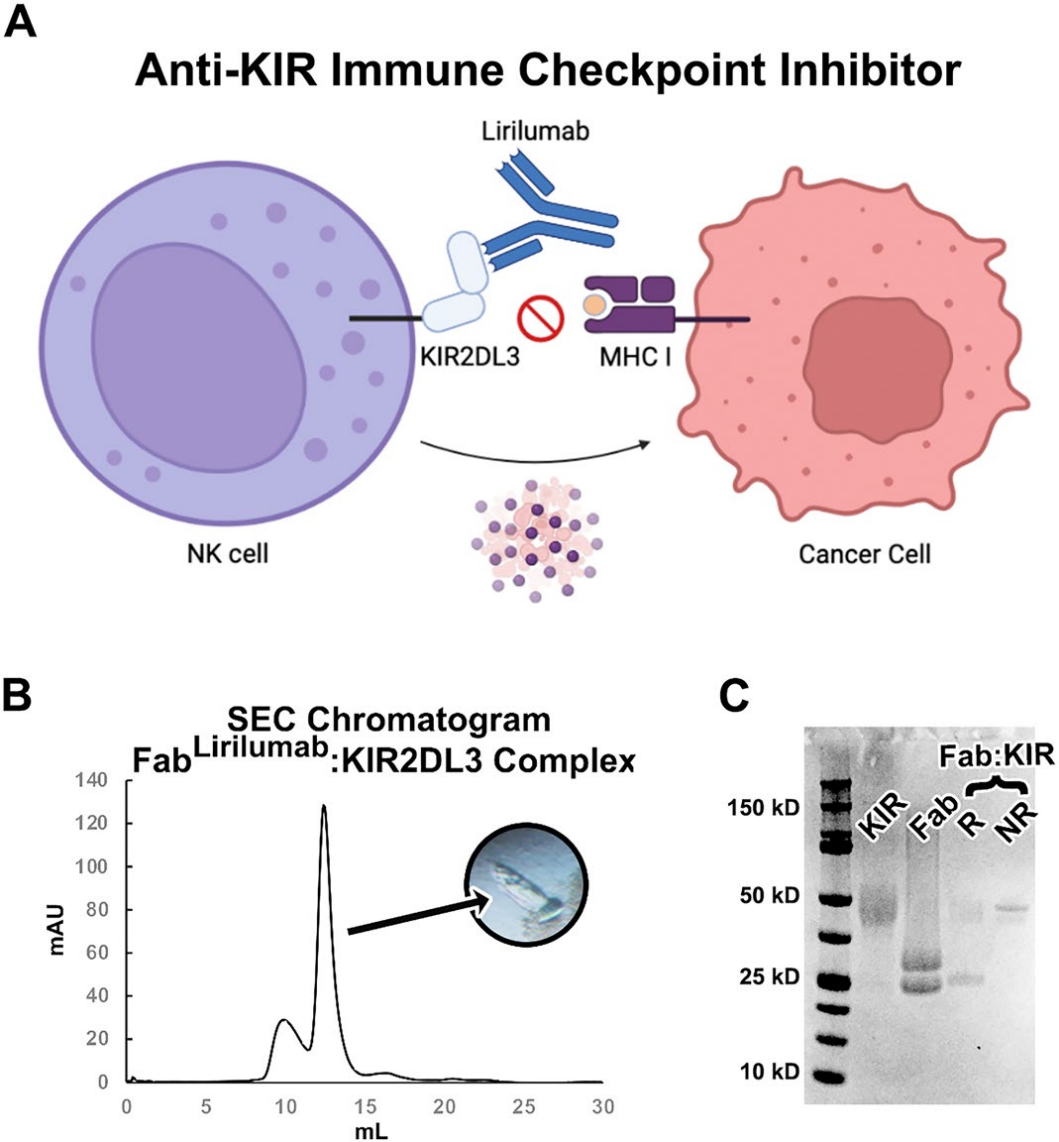
The immune checkpoint inhibitor lirilumab binds to NK cell inhibitory KIR receptors. (**A**) Graphical summary describing the mechanism of the immune checkpoint inhibitor antibody lirilumab targeting inhibitory KIR receptors on NK cells, which promotes NK cell-mediated clearance of cancer cells. Panel made with BioRender. (**B**) Size exclusion chromatography purification and crystal formation of the Fab^Lirilumab^—KIR2DL3 complex. (**C**) Coomassie-stained SDS-PAGE of KIR2DL3 (KIR), Fab^Lirilumab^ in a reducing buffer (Fab), and the purified Fab^Lirilumab^—KIR2DL3 complex (Fab:KIR) in a reducing (R) or non-reducing (NR) buffer.

Some of the first evidence that blocking KIR function could improve cancer outcomes came from hematopoietic stem cell transplants in leukemia patients where there was a KIR:HLA repertoire mismatch – patients receiving transplants with an alloreactive NK cell population showed improved disease-free survival rates and reduced rate of relapse^15,16^. Early in vitro experiments demonstrated that KIR-targeted antibody fragments could promote NK-mediated cytotoxicity toward transfected tumor cell lines expressing HLA Cw3^17^. In mouse models, blocking the mouse KIR receptor equivalent, Ly49, led to NK cell dependent clearance of RMA lymphoma cells but not syngeneic B6 spleen cells^18^. Data like these led to interest in the development of anti-KIR monoclonal antibody therapeutics to improve tumor clearance by blocking signaling of inhibitory KIR receptors on NK cells (Fig. 1A)^19,20^.

Lirilumab (IPH2102, descended from 1-7F9) is a monoclonal antibody therapeutic candidate that was developed to target the two primary inhibitory KIRs (KIR2DL1 and KIR2DL2/3) (Fig. 1A). To obtain such an antibody, mice bearing human genomic IgG loci were immunized with BW5417 cells expressing KIR2DL1, followed by 3 boosters with the extracellular region of recombinant KIR2DL3^21^. The monoclonal antibody selected for development demonstrated high affinity binding to KIR2DL1 and -L3, weaker binding to KIR2DS4, and no binding to the divergent KIR2DL4 or any KIR3D receptor^19,20^. The variable domains of the anti-KIR antibody were cloned into an IgG4 constant region framework to avoid antibody dependent cytotoxicity toward NK cells^19^. In preclinical studies, lirilumab enhanced in vitro lysis of patient-derived acute myeloid leukemia (AML) blasts when administered with IL-2 activated, HLA-matched, NK-cells. Similarly, NOD-SCID mice challenged with both AML cells and NK cells were rescued by lirilumab where control mice succumbed to leukemia^21^. Phase I clinical trials demonstrated initial safety of lirilumab, prompting a number of phase II clinical trials using lirilumab alone and in combination with other chemotherapeutics^21–24^. While lackluster efficacies of lirilumab in these trials have likely hindered its advancement into phase III clinical trials, a lack of understanding of lirilumab’s epitope and specificities for different KIR variants and usages in different patients may be preventing additional phase II clinicals in more stratified patient populations.

Here we determined the crystal structure of the lirilumab variable domains bound to the inhibitory KIR2DL3. The lirilumab epitope overlaps with the binding site for HLA, revealing the mechanism of inhibition by lirilumab. Further investigation of the lirilumab epitope reveals several key amino acids that vary across the human population-level KIR repertoire, and binding studies demonstrate the importance of these amino acids for lirilumab binding. Altogether, our data support the further development of lirilumab and other KIR-targeting immune checkpoint inhibitors.

## Results

**Figure 2:**
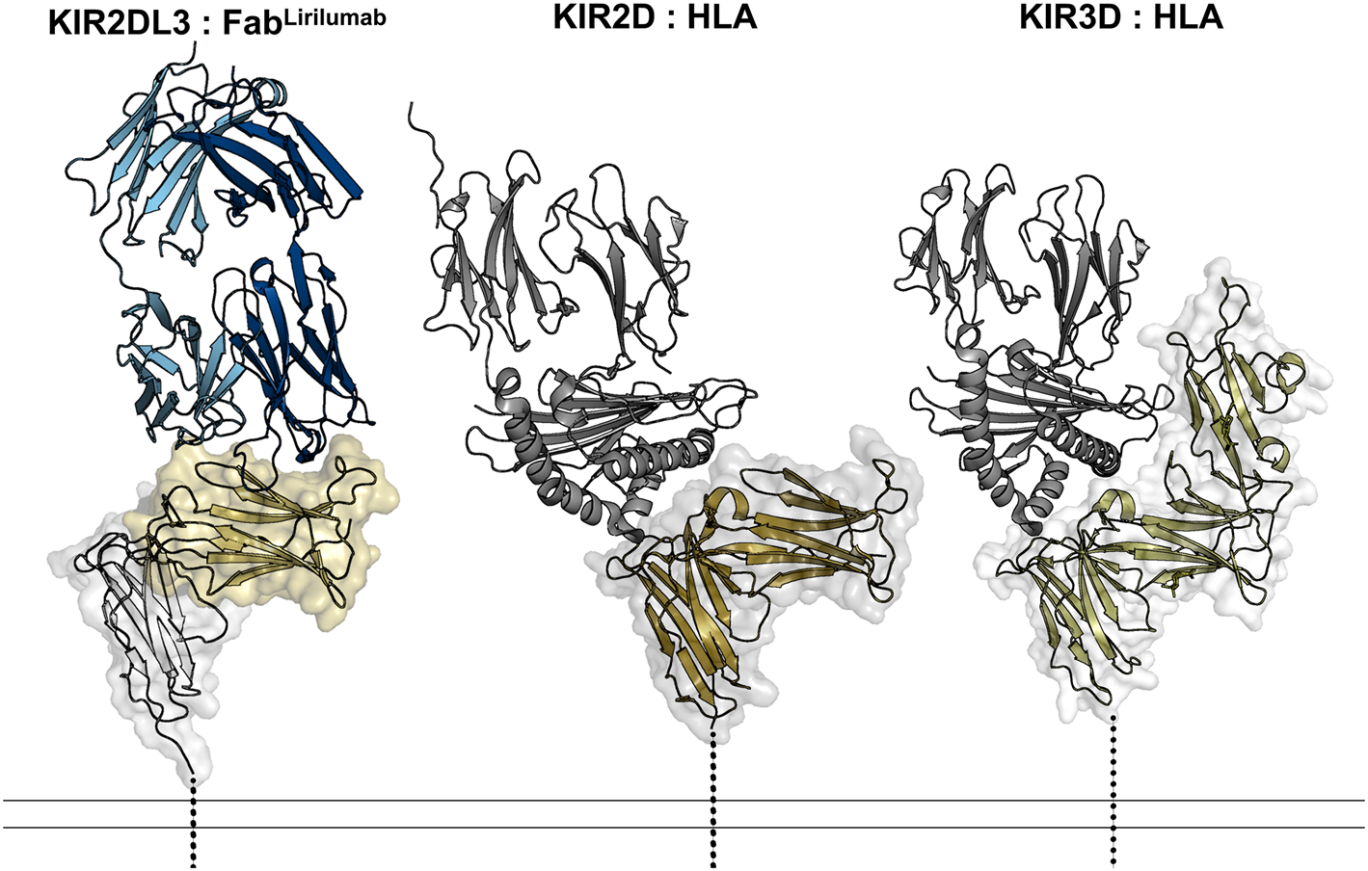
The lirilumab epitope overlaps with the HLA binding site but is not exposed on KIR3D class receptors. (Left) KIR2DL3 bound to Fab^Lirulumab^, with the Fab^Lirulumab^ heavy chain colored navy, light chain colored light blue, KIR2DL3 domain 1 colored gold and domain 2 colored white. (Center) KIR2DL1 bound to HLA-Cw4 (PDB 1im9)^38^, with HLA colored gray and KIR2DL1 colored bronze. (Right) KIR3DL1 bound to HLA-B*57:03 (PDB 6v3j)^39^, with HLA colored gray and KIR3DL1 colored olive. Dashed lines represent the locations of transmembrane domains. There is an approximate 134 Å^2^ overlap between the lirilumab epitope and the HLA binding site, revealing how lirilumab can block HLA binding. Also, these structures reveal that the lirilumab epitope is occluded by the additional domain of KIR3D receptors, explaining why lirilumab does not bind KIR3D receptors.

To define the epitope of lirilumab, we first produced a recombinant antigen binding fragment (Fab) encoding the variable domains of lirilumab fused to the constant domains of a human IgG1 Fab. We termed this antibody construct Fab^Lirilumab^. We also produced the recombinant ectodomain of KIR2DL3, which is reported to bind lirilumab with high affinity^19,20^. A complex of Fab^Lirilumab^ and KIR2DL3 was purified by size exclusion chromatography and the presence of intact KIR2DL3 and Fab^Lirilumab^ was confirmed by SDS-PAGE (Fig. 1B,C). The Fab^Lirilumab^—KIR2DL3 complex was screened in crystallization trials and after ~ 3 weeks crystals were obtained. X-ray diffraction data were collected and molecular replacement was used to determine the crystal structure of the Fab^Lirilumab^—KIR2DL3 complex at 2.75 Å resolution (Fig. 2, Table 1). During molecular replacement, a solution could only be obtained when the KIR2DL3 structure was split into its two separate Ig-like domains. The final structure is a complex of Fab^Lirilumab^ with only domain 1 of KIR2DL3 (Fig. 2), and analysis of the crystallographic packing reveals that the other domain could not fit. Thus, we suspect that proteolysis of the complex occurred during crystallization trials and the extended time required for crystallization. Nevertheless, the resulting structure from the crystallized complex enabled detailed analyses of the lirilumab epitope.

**Table 1:** Crystallography data collection and refinement statistics.

Data collection	Fab^lirilumab^: KIR2DL3 complex (PDB ID 8TUI)
Space group	P12_1_1
a, b, c (Å)	58.38, 84.27, 62.27
α, β, γ (°)	90.00, 115.86, 90.00
Resolution (Å)^a^	50.00–2.75 (2.80–2.75)
*R*_merge_ (%)^a^	0.138 (0.920)
Mean I/σ(I)^a^	14.0 (1.7)
Completeness (%)^a^	98.5 (98.1)
Redundancy^a^	6.5 (5.9)
CC_1/2_	0.94 (0.77)
Refinement	
Resolution (Å)	44.58–2.751
Number of reflections	13,574
*R*_work_/*R*_free_^b^	0.2112/0.2548
Atoms	4176
Protein	4086
Water	85
Ligand	5
Mean B factor (Å^2^)	68.5
Protein	67.96
Water	45.98
Ligand	95.76
R.m.s deviation	
Bond lengths (Å)	0.008
Bond angles (°)	1.13
Ramachandran (%)	
Favored	94.9
Allowed	5.1
Outliers	0

^a^The values in parentheses are for the outermost shell.

^b^*R*_free_ is the R-factor calculated with the  5% of the data excluded from the refinement.

To understand the mechanism of inhibitory action by lirilumab, we compared the Fab^Lirilumab^—KIR2DL3 structure to known HLA–KIR complex structures (Fig. 2). While the lirilumab epitope is limited to domain 1 of KIR2DL3 (Fig. 2, left), the epitope overlaps with approximately 134 Å^2^ of the binding site for HLA, which spans both domains 1 and 2 of KIR2DL3 (Fig. 2, center). Thus, lirilumab directly blocks the binding site for HLA. We further sought to understand why lirilumab does not bind to KIR3D receptors, despite the significant homology between KIR2DL3 and some KIR3D receptors. Comparison to a structure of HLA bound to a KIR3D receptor reveals that lirilumab’s epitope is wholly obscured by the third Ig-like domain in KIR3Ds (Fig. 2, right), explaining its inability to bind receptors in that class.

**Figure 3:**
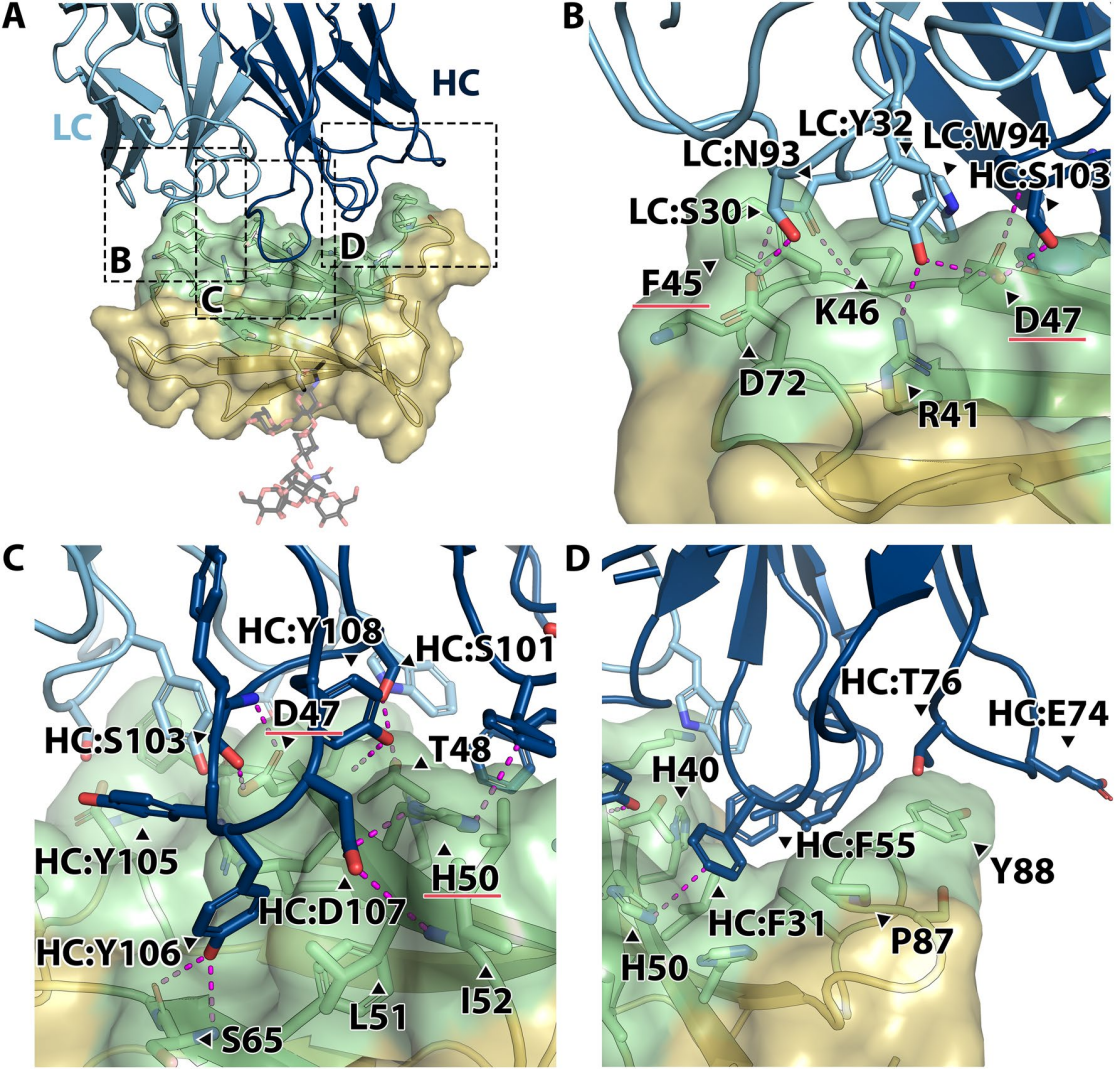
Intermolecular interactions at the lirilumab:KIR2DL3 interface. (**A**) Interface of the Fab^Lirilumab^—KIR2DL3 complex, with the Fab^Lirulumab^ heavy chain colored navy, light chain colored light blue, KIR2DL3 domain 1 colored gold, and KIR2DL3 epitope amino acids colored green. The KIR2DL3 N-glycosylation site on N63 is shown as sticks. Dashed boxes highlight the location of close-up panels (**B**)–(**D**). (**B**) Close-up view of lirilumab light chain interactions with KIR2DL3, with notable hydrogen bonding interactions across the interface. (**C**) Close-up view of lirilumab heavy chain CDR-H3 loop interactions with KIR2DL3. Note the hydrogen bonding and hydrophobic interactions by the loop residues 103–108 (SYYYDY). (**D**) Close-up view of lirilumab heavy chain CDR-H1 and -H2 loop interactions with KIR2DL3, which are mostly hydrophobic. In panels (**B**), (**C**), hydrogen bonds are denoted by magenta dashed lines.

We further investigated the molecular features that govern lirilumab’s interactions with KIRs (Fig. 3). Lirilumab binds to a 954 Å^2^ conformational epitope on domain 1 of KIR2DL3 that is distal from its single N-linked glycosylation site (Fig. 3A). No significant structural changes to KIR2DL3 are observed when in complex with lirilumab compared to its structure alone (RMSD of 0.7 Å^2^ and TM-score of 0.96 for KIR2DL3 domain 1 structural alignments)^25^. Five CDR loops of lirilumab interact with KIR2DL3, however the heavy chain CDR loops account for the majority of the epitope footprint, covering 659 Å^2^ of the binding interface. In the light chain, CDR-L1 and -L3 interact with KIR2DL3, making up 295 Å^2^ of the epitope.

Many of the key interactions at the lirilumab:KIR2DL3 interface form an extensive hydrogen bond network. Lirilumab’s light chain S30 and Y32 from CDR-L1 and N93 from CDR-L3 form hydrogen bonds with KIR side chains D72, R41, and D47, as well as K46 backbone (Fig. 3B). Lirilumab’s heavy chain S101 and S103 from CDR-H3 form hydrogen bonds with KIR2DL3 side chains T48 and D47, respectively (Fig. 3C). Also, lirilumab’s D107 from CDR-H3 forms hydrogen bonds with KIR2DL3 side chain H50 and I52 backbone (Fig. 3c). Finally, two tyrosines, Y106 and Y108, in a tyrosine rich heavy chain CDR-H3 along with Y105, form hydrogen bonds with KIR S65 and T48 backbones, respectively (Fig. 3C).

In addition to this hydrogen bond network, there are a number of hydrophobic and pi-stacking interactions at the lirilumab:KIR2DL3 interface. First, lirilumab’s heavy chain CDR-H1 and -H2 loops nestle within a KIR groove and form pi-stacking interactions between F31 and KIR H50 as well as between F55 and KIR H40 (Fig. 3D). Interestingly, lirilumab’s heavy chain CDR-H1 and -H2 loops as well as the heavy chain framework region 3 loop containing E74 and T76 also interact with KIR P87 and Y88 (Fig. 3D). The heavy chain CDR-H3 Y106 tucks into a hydrophobic pocket formed by KIR L49, L51, and F64 (Fig. 3C). Finally, KIR F45 side chain lies between the lirilumab CDR-L1 and -L3 loops while KIR K46 forms a cation-pi interaction with W94 from CDR-L3 (Fig. 3B).

**Figure 4:**
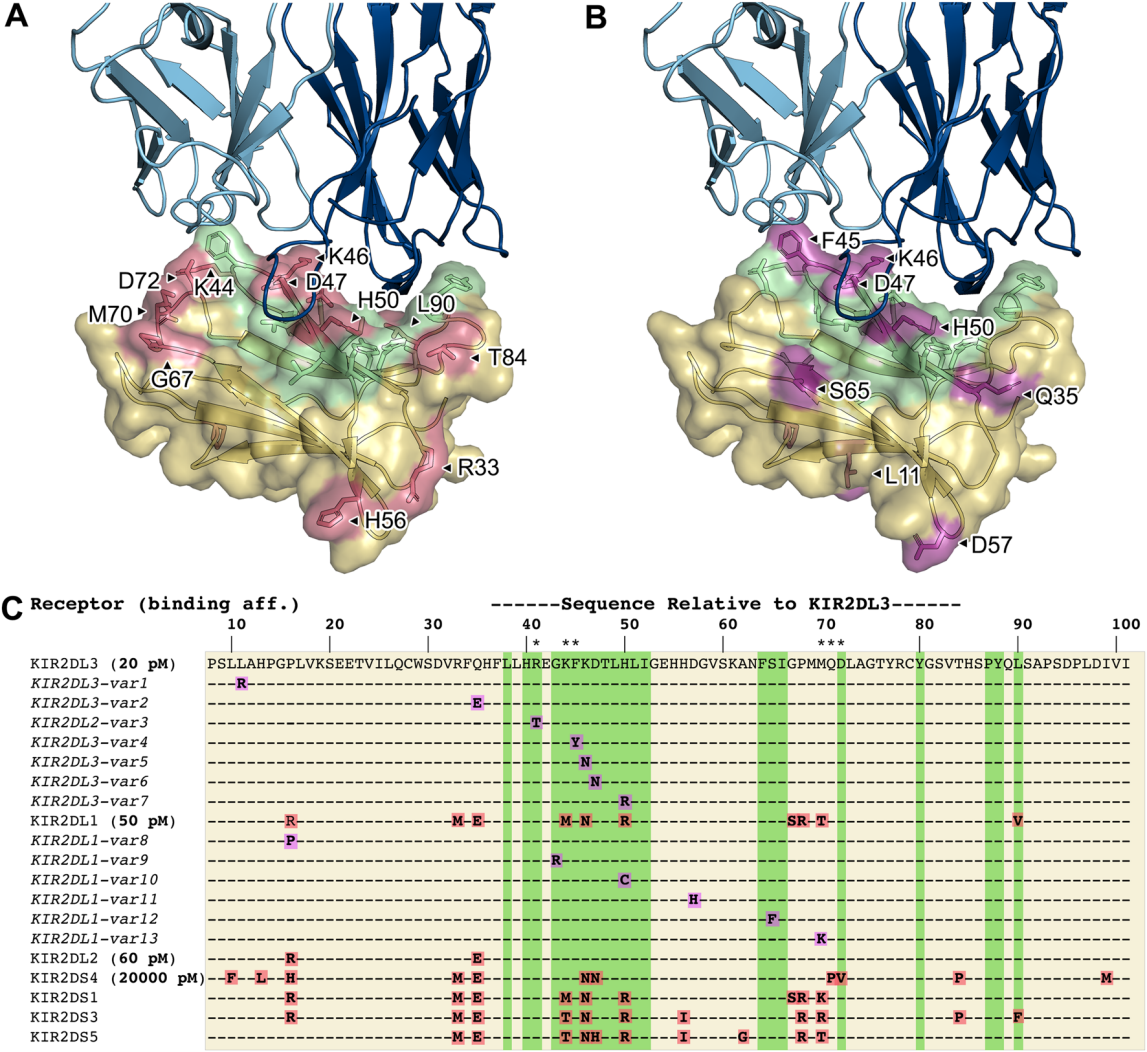
Polymorphism between KIR2D class receptors and their variants is often part of the lirilumab epitope. (**A**), (**B**) Lirilumab-similar binding footprint (green) overlaid with polymorphic sites between KIR2D class receptors (red) and sites of KIR2DL3 and KIR2DL1 variant polymorphisms (magenta). (**C**) A sequence alignment of KIR2D receptors and variants (from EMBL-EBI IPD-KIR database, release 2.12) that result in amino acid mutations in the KIR domain bound by lirilumab. KIR2DL1 and KIR2DL2 variant mutations are displayed relative to their respective parent receptor sequences, while mutations in all other aligned sequences are relative to KIR2DL3. Sequence numbering corresponds to the KIR2DL3 sequence. Asterisks (*) above the sequence alignment indicate amino acids involved in KIR:HLA binding. Alignment coloring corresponds to the surface colors in panels A and B. Binding affinity constants as previously determined by ELISA (Table 2) are shown in bold font next to the KIR2D receptor name. See Table S2 for variant accession numbers and listing of other high frequency KIR2D variants.

To understand the structural basis for lirilumab binding to other inhibitory KIRs, we performed a sequence alignment of KIR2D class receptors and mapped these differences onto the structure (Fig. 4). This alignment revealed an intriguing overlap between the lirilumab:KIR interface and the occurrence of amino acid variation in distinct KIRs (Fig. 4C). First, comparison of sequences from different KIR2D class receptors along with literature-reported binding affinities with lirilumab identifies several amino acids that may affect affinity (Fig. 4A,C and Table 2)^20^. For example, KIR2DL1 has three amino acid differences in the lirilumab epitope (K44 to M44, K46 to N46, and H50 to R50, in KIR2DL3 numbering) yet it has only 2-3-fold lower affinity for lirilumab compared to KIR2DL3, suggesting that those sequence differences do not significantly affect affinity (Fig. 4C and Table 2). Indeed, the structure reveals that these amino acids are on the periphery of the epitope and could accommodate these side chain differences (Fig. 4A). On the other hand, KIR2DS4 also has three amino acid differences in the lirilumab epitope (K46 to N46, D47 to N47, and D72 to V72, in KIR2DL3 numbering), yet it has a ~ 1000-fold lower affinity for lirilumab compared to KIR2DL3. One difference, K46 to N46, is found in KIR2DL1 and is therefore unlikely to significantly affect affinity (Fig. 4A). D72 is on the periphery and makes a hydrogen bond with lirilumab’s light chain S30 from CDR-L1, and while the change to valine in KIR2DS4 would lose this bond, it is also unlikely to significantly affect affinity. In contrast, D47 is in the center of the epitope, is completely masked by lirilumab, and makes a number of hydrogen-bond interactions with lirilumab (Fig. 3B,C). Thus, a difference of D47, a negatively-charged hydrogen-bind-acceptor side chain, to asparagine, an uncharged polar side chain with hydrogen-bond donor and acceptor capabilities, and disruption of hydrogen bond networks, is the most likely reason for the ~ 1000-fold lower affinity of lirilumab for KIR2DS4.

**Table 2:** Binding affinities between Lirilumab (IgG1) and KIR receptors.

	K_D_ (nM)		
	*SPR*^*a*^	*ELISA*^*b*^	*BLI*^*c*^
KIR2DL1	10.3	0.05	10.0^d^
KIR2DL2		0.06	–
KIR2DL3	3.5	0.02	2.8
KIR2DS4		20	–
KIR2DL3 F45Y			9.9
KIR2DL3 D47N			1650^e^
KIR2DL3 H50R			11.2

^a^Surface plasmon resonance (SPR) values reported in^19^ using immobilized antibody 1-7F9 and KIR2D class receptor as the analyte.

^b^ELISA K_D_ values as reported in^20^. KIR2D class receptors were adhered to the ELISA plate, after which lirilumab was added at different concentration, allowing for enhanced binding by avidity. KIR2DS4 measurement was performed with a KIR2DS4-Fc fusion construct.

^c^Except where otherwise noted, K_D_s were determined by global fitting of a 1:1 langmuir binding model to two-fold dilution series data. Values reported are the average of 3 technical replicates.

^d^Commercially sourced KIR2DL1 (Sino Biological 13,145-H08H).

^e^K_D_ constant determined by steady state model. In samples where a 1:1 binding model fit well, steady state K_D_ and Langmuir-derived K_D_ were near-equivalent values.

To evaluate population level diversity between KIR variants, we performed a sequence alignment of non-synonymous variants within KIR2D class receptors and mapped these differences onto the structure (Fig. 4, for additional variants and their frequencies see Supplementary Table S2). This alignment again revealed an intriguing overlap between the lirilumab:KIR interface and the occurrence of amino acid variation in distinct KIRs (Fig. 4C)^26^. First, we observed a KIR2DL3 D47N variant, which is the same amino acid change that we hypothesize is the reason for the greatly reduced affinity of KIR2DS4 for lirilumab (described above)(Fig. 4B). In addition, there is a KIR2DL3 F45Y variant, which would extend the phenyl ring with a hydroxyl group that we hypothesize could affect binding by clashing or forcing a shift of the nearby S28 on the lirilumab CDR-L1 (Fig. 4B). Finally, we observed a KIR2DL3 H50R variant. H50 interacts with residues in lirilumab’s CDR-H1 and -H3 loops (Figs. 3C, 4B), and while change to an arginine is not expected to sterically clash, it may affect these hydrogen bonding and pi-stacking interactions with lirilumab.

**Figure 5:**
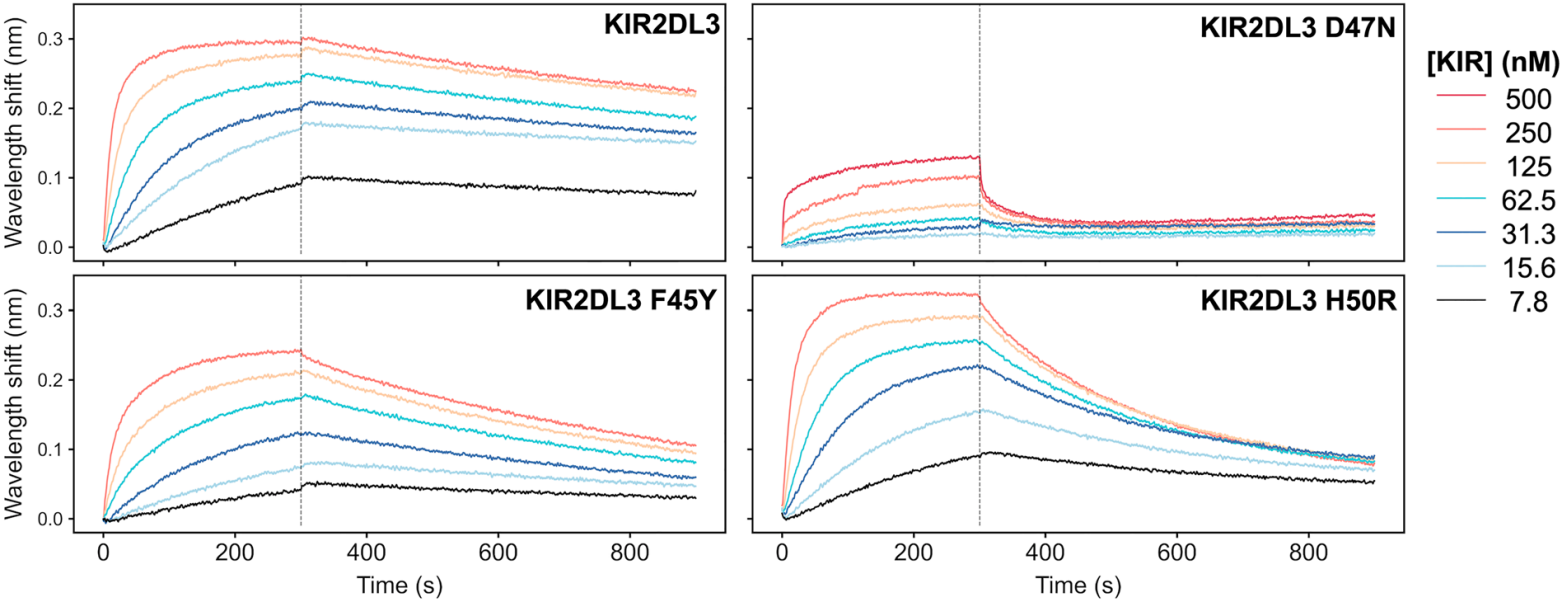
The high-affinity interaction between lirilumab and KIR2DL3 is affected by polymorphisms present in KIR2DL3 variants and in KIR2DS4. Biolayer interferometry kinetics plots are shown for lirilumab (IgG1) and three KIR2DL3 mutants in neutral pH conditions. In each assay, IgG1 lirilumab was loaded onto AHC sensor tips and equilibrated to assay buffer prior to analyte binding. Each data series consists of 6 two-fold dilutions starting at an analyte concentration of 250 nM (except KIR2DL3 D47N, where the peak concentration is 500 nM). Representative plots from among three assay replicates are shown.

To test these observations, we produced several KIR2DL3 proteins with variant mutations in the lirilumab epitope and tested them for binding to lirilumab (IgG1) utilizing biolayer interferometry (BLI) (Fig. 5, Supplementary Fig. S1, Table S1). Dissociation constants are compared to reported values (Table 2)^19,20^. By BLI, a dissociation constant (K_D_) of 2.8 nM was determined for the interaction of lirilumab (IgG1) and wild-type KIR2DL3, which is consistent with the published value of 3.5 nM determined by surface plasmon resonance (SPR). In contrast, the affinity determined for the interaction of lirilumab (IgG1) and KIR2DL3 D47N was dramatically reduced, with a K_D_ of 1650 nM and a notably faster off-rate (Table 2, Fig. 5). Thus, the single functional group change from carboxylic acid to amide dramatically disrupted the lirilumab epitope. In addition, both the KIR2DL3 F45Y and H50R variant mutations modestly weakened antibody binding by 3-4-fold (Table 2, Fig. 5). The lirilumab affinity for KIR2DL3 H50R is consistent with the affinity for KIR2DL1, which also contains the H50R mutation (Table 2, Fig. 5). While our data are very similar to published SPR affinities, we note that there are significant differences with published ELISA affinities (Table 2). This difference is most likely due to differences between methodologies, with ELISA experiments performed by coating plates with KIR proteins and binding with lirilumab antibody, resulting in lower K_D_ values due to contributions of antibody avidity. Nevertheless, our BLI data are consistent to the trends in ELISA data, whereby KIR2DL3 has the highest affinity, and the KIR2DL3 D47N variant mutation that is also found in KIR2DS4 results in a dramatically reduced affinity for lirilumab.

## Discussion

In therapeutic targets with significant population-level genetic diversity, the structural determination of binding epitopes is an important consideration, allowing more precise administration of therapeutics when paired with patient genome profiling, or prioritization during the discovery stage, enabling selection of a more broad-spectrum therapeutic candidate. The KIR family of receptors are relatively variable and an individual’s NK cells may include receptors bearing polymorphisms or may only express a subset of receptors from an individual’s repertoire^11^. This variability could result in a situation where therapeutic antibodies targeting KIRs may not be equally effective for all individuals. Here, we have defined the epitope for lirilumab, a pan-KIR2D immune checkpoint inhibitor currently in phase II clinical trials, and we have uncovered KIR2D variants that may influence its clinical efficacy.

We focused our binding studies on variants within the primary inhibitory KIRs, KIR2DL1 and KIR2DL2/3, that fall within the epitope of lirilumab. First, we found that the KIR2DL3 D47N variant has a dramatic > 500-fold weaker affinity for lirilumab compared to the wild-type KIR2DL3, suggesting that individuals with this variant may respond differently to lirilumab therapy. While the D47N mutation is rare (6 in 1000 in East Asian population), it is an important reminder of how an individual’s genomic variability could influence therapeutic outcomes (see Table S2 for a list of inhibitory KIR variants and global population frequencies). We also investigated the KIR2DL3 F45Y and H50R variants that are much more common (5–15% of the population overall, respectively, with higher frequencies in some sub populations), and we observed 3-4-fold reductions in affinity for lirilumab, which may also be able to influence patient response to lirilumab therapy^27^. Although there are several other inhibitory KIR2D variants within the lirilumab epitope that could be explored, such as KIR2DL3 R41T, K46N, KIR2DL1 G43R, R50C, S65F, and T70K (all in KIR2DL3 numbering) (Fig. 4c), our structural observations suggest that these variants will have modest or no effects on lirilumab binding. In addition to polymorphisms directly within the lirilumab epitope, there is evidence that mutations distal to the epitope site can affect KIR:HLA binding, putatively through changes in secondary and tertiary structure, indicating distal variants may warrant additional investigation^28^.

Despite demonstrating safety in pre-clinical and phase I clinical trials, phase II trials involving lirilumab often did not demonstrate significant improvement over the standard of care (clinical trial IDs NCT02399917, NCT01687387, NCT01592370)^29^. Our studies support that patient genome profiling for inhibitory KIR receptor variation may enable patient stratification for individuals with a matching lirilumab epitope and improve outcomes. Our structure also provides a roadmap to optimize lirilumab affinity and/or specificity and alter clinical outcomes. For example, affinity maturation of the lirilumab light chain CDR-L1 and -L2 loops may increase affinity and epitope overlap with the HLA binding site (Fig. 2). However, we caution that a higher-affinity anti-KIR antibody could be detrimental, as there is evidence of inhibitory receptor down-regulation when KIRs are continuously occupied by an antibody and unable to signal^10^. This prolonged KIR blockade and reduction in KIR expression may result in NK cell anergy^10^. One potential solution is to combine anti-KIR antibody therapy with stimulatory cytokines^10^. Another possible solution that is enabled by our studies is the engineering of lirilumab to have a weaker affinity, thereby increasing NK cell’s cytotoxic activity while still allowing intermittent HLA sampling by inhibitory KIRs. Engineered versions of lirilumab, as well other anti-KIR antibodies with differing affinities and KIR specificities, could be used to explore these hypotheses further in pre-clinical studies^20^.

The clear importance of NK cell activity in tumor clearance and disease-free survival rates remains a strong motivator to develop therapeutics that can modulate NK cell activity. Our studies reveal general concepts for the development of inhibitory KIR-targeting immune checkpoint inhibitors, most notably epitope evaluation and deep investigation of population scale variation, which could lead to improved clinical outcomes through precision application of next generation antibody therapeutics.

## Methods

### Study Design

This study was performed to investigate the epitope of the KIR-blocking monoclonal antibody lirilumab to support improved therapeutic administration parameters and/or future development of novel antibodies that may have different or improved therapeutic characteristics. We hypothesized that some feature of the lirilumab epitope may be contributing to its underwhelming performance in stage II clinical trials, either through inadequate blocking of the HLA binding site or overlap with disruptive polymorphic sites present in patients’ inhibitory KIR receptor repertoire. In vitro binding experiments involved technical replicates performed in triplicate. Various Fab^lirilumab^—KIR2DL3 complexes were subjected to crystallographic screening under the assumption that molecular replacement methodologies would allow structure determination, as structures of KIR2DL3 and human antibody Fab fragments had been solved previously. The successful structure determination then informed design of KIR receptor mutants and subsequent kinetics assays. Experiments were not blinded and experimenters were aware of experimental conditions.

### Expression and purification of recombinant KIR2DL3

The expression plasmid for recombinant KIR2DL3 is a derivative of pcDNA 3.1 containing a CMV promoter, CCR5 signal sequence, and a codon-optimized gene encoding the human KIR2DL3 ectodomain (residues 1–224, RefSeq: NP_056952.2) fused to a C-terminal c-Myc tag and a 6-histidine tag, similar to that expressed by Maenaka et al.^30^. Mutant KIR plasmids were generated by site-directed mutagenesis. The plasmids were verified by Sanger sequencing. Sterile, endotoxin free expression plasmid DNA was produced using the Invitrogen Plasmid Maxiprep Kit (Invitrogen A31231).

CHO-S (Thermo Fisher R80007) cells were electroporated in the presence of the KIR2DL3 expression plasmid using a MaxCyte electroporator with OC-400 electroporation cuvettes (MaxCyte SOC4) following the manufacturer’s instructions, enabling transient expression of the recombinant KIR2DL3 protein. After transfection, cells were grown with OptiCHO Expression media (Thermo Fisher 12,681,011, supplemented with 8 mM L-glutamine, 0.1 mM Sodium Hypoxanthine, 16 µM thymidine) in shake flasks using a Khuner shaker incubator at 32 °C, 8% CO2, and 85% humidity. The day after transfection, filter sterilized sodium butyrate was added to the cell culture media to a final concentration of 1 mM. Each day after transfection, cells were fed with 3% volume of an enriched feed media (containing 7 mM L-glutamine, 5.5% glucose, and 23.4 g/L yeastolate in CHO CD Efficient Feed). Approximately six days after electroporation (or when cell viability drops below 60%), media and cells were centrifuged at 6000 g for 10 min and the supernatant was decanted and 0.22-micron filtered.

An appropriate volume of Elution buffer (20 mM MOPS, 150 mM NaCl, 200 mM imidazole, pH 7.5) was added to the filtered CHO-S supernatant to bring total imidazole concentration to 10 mM. Using an AKTA Pure FPLC instrument, a HisTrap FF column (Cytvia 17,531,901) was equilibrated with Wash buffer (20 mM MOPS, 150 mM NaCl, 20 mM imidazole, pH 7.5), and the CHO-S supernatant was loaded, washed with 10 column volumes (CV) Histidine Wash buffer, and KIR2DL3 protein was eluted using a 5 CV linear gradient to 100% Elution buffer. The elution was monitored by absorbance at 280 nm and the elution fractions corresponding to the 280 nm peak were assessed by SDS-PAGE. The purest elution fractions were combined, concentrated in a 10kD spin concentrator, and dialyzed into 20 mM MOPS, 150 mM NaCl pH 7.5.

### Expression and purification of recombinant lirilumab (IgG1) and Fab^Lirilumab^

Synthetic cDNA encoding the heavy and light chain variable regions of lirilumab (GSRS UNII identifier S9XDI9W918) was cloned by Gibson Assembly into the pCMV-VRC01 antibody heavy and light chain plasmids, in place of the variable regions of antibody VRC01, a human anti-HIV IgG1 antibody targeting the gp120 protein^31^. For the Fab^Lirilumab^ heavy chain, only the variable region and the constant heavy 1 region ending with residues DKKVEPKSC was included, followed by an alanine-serine linker, C-terminal thrombin cleavage site, and a Twin Strep-tag. All antibody sequences were in-frame with an N-terminal signal sequence. The plasmids were verified by Sanger sequencing. Sterile, endotoxin free expression plasmid DNA was produced using the QIAGEN Plasmid Maxi Kit (QIAGEN Cat. No. 12163).

HEK 293F Freestyle (ThermoFisher R79007) cells were electroporated in the presence of the lirilumab heavy and light chain expression plasmids using a MaxCyte electroporator with OC-400 electroporation cuvettes (MaxCyte SOC4) following the manufacturer’s instructions. After transfection, cells were grown in Freestyle 293 Expression media (Gibco 12,338–018) in vented shake flasks using a Khuner shaker incubator at 37 °C, 8% CO2, and 85% humidity. Four days after electroporation, media and cells were centrifuged at 6000 g for 10 min and the supernatant was decanted and 0.22-micron filtered.

For purification of lirilumab (IgG1), filtered supernatant was incubated with protein A resin (Pierce 20,334), eluted with glycine pH 3.1, and immediately neutralized with 1 M Tris pH 9.0. Lirilumab (IgG1) was dialyzed into phosphate buffered saline pH 7.4 and purity was assessed by SDS-PAGE.

For purification of Fab^Lirilumab^, filtered 293F supernatant was diluted 1:1 with Strep Wash buffer (20 mM MOPS, 150 mM NaCl, 1 mM EDTA, pH 7.5) and 1.6 mL of Biolock (IBA Life Sciences 2–0205-050) was added per liter of 293F media. The diluted supernatant was loaded onto a StrepTrap column (GE Healthcare 28–9075-47) equilibrated with Strep Wash buffer, the column was washed with 10 CV Strep Wash buffer, and the Fab^Lirilumab^ was eluted with 8 CV Elution buffer (20 mM MOPS, 150 mM NaCl, 1 mM EDTA, 2.5 mM desthiobiotin, pH 7.5). Eluant fractions with high 280 nm absorbance were combined, concentrated in a 10kD spin concentrator, and dialyzed into 20 mM MOPS, 150 mM NaCl pH 7.5.

### Structure determination of the Fab^Lirilumab^—KIR2DL3 complex

Purified, dialyzed Fab^Lirilumab^ and KIR2DL3 were combined in a 1.2:1 molar ratio and incubated at 4 °C for 1 h to allow complex formation. The mixture was injected onto a gel filtration column (Superdex 200 10/300 GL, GE Healthcare 28–9909-44) pre-equilibrated in 20 mM MOPS, 150 mM NaCl pH 7.5 and fractions were collected. The contents of fractions were assessed by SDS-PAGE, and fractions containing both Fab^Lirilumab^ and KIR2DL3 were pooled and concentrated in a 10kD spin concentrator to 7.5 mg/ml.

A sitting drop crystal screen was set up using the concentrated Fab^Lirilumab^—KIR2DL3 complex where each drop contained a 1:1 mixture of the complex and the screening condition buffer. After several weeks crystals were observed in a condition containing 200 mM Potassium Sodium Tartrate and 20%(w/v) PEG 3350. The crystal was transferred to a cryoprotectant solution of 0.2 M potassium sodium tartrate, 20% (w/v) PEG 3350, 6% ethylene glycol, 6% DMSO, and 6% glycerol and then flash frozen in liquid nitrogen. Diffraction data were collected at cryogenic temperature at the Advanced Photon Source on beamline 23-ID-D using a wavelength of 1.03 Å.

Diffraction data from a single crystal were processed with HKL2000^32^. The Fab^Lirilumab^—KIR2DL3 complex structure was solved by molecular replacement using a Fab structure extracted from PDB 6XOC^33^ and KIR2DL3 domains extracted from PDB 1B6U^30^ with the PHASER^34^ program. The structure was then partially rebuilt with the auto-build program within the PHENIX suite and completed manually using Coot followed by refinement using PHENIX.refine and Coot^35,36^.

### Biolayer interferometry (BLI) binding assays

Wild-type and mutant KIR proteins used in biolayer interferometry (BLI) assays were produced similarly to the methods described above, except they were expressed in HEK 293F cells instead of CHO-S cells. BLI assays were performed with an Octet RED384 instrument with temperature set to 25 °C and shaking at 1000 rpm for all assays. The assay buffer used was composed of 20 mM MOPS (pH 7.5 at 22 °C), 150 mM NaCl, 0.1% BSA, and 0.02% Tween-20. Anti-human IgG biosensors (AHC) were hydrated in assay buffer for at least 10 min before the start of each BLI experiment. Assays were performed in either black tilted-bottom 384-well plates or black flat-bottomed 96-well plates which using 40 µL or 200 µL of sample, respectively. To determine binding affinities of lirilumab (IgG1) for each KIR receptor protein, BLI experiments were performed as follows: (1) pre-hydrated AHC biosensors were dipped into assay buffer for 120 s to establish a baseline; (2) biosensor were dipped into 7.5 µg/mL lirilumab (IgG1) in assay buffer for 60 s to load the antibody onto the biosensors; (3) biosensors were dipped into assay buffer for 60 s to confirm stable antibody loading and establish a new baseline; (4) lirilumab (IgG1)-loaded biosensors were dipped in wells containing a concentration series of KIR protein in assay buffer for 300 s to determine the association rate (5) biosensors are dipped in assay buffer for 600 s to determine the dissociation rate. No-KIR-antigen and no-antibody controls were performed so baseline signal drift could be accounted for and to confirm that non-specific binding does not occur between antigens and biosensors. A global association 1:1 model was used to fit at least 4 curves per replicate to determine the on- and off-rates and calculate the dissociation constant (K_D_). All binding assays were performed in technical triplicates, and the average K_D_ of the three replicates are reported in Table 2. Curve fitting was unsuccessful for the KIR2DL3 D47N mutant, preventing K_D_ determination with on- and off-rates. Instead, a steady state equilibrium model was utilized.

### Sequence alignment of KIR receptors and variants

Protein sequences used in the KIR receptor alignment were downloaded from RefSeq’s non-redundant protein database: KIR2DL1_(NP_055033.2), KIR2DL2_(NP_055034.2), KIR2DL3_(NP_056952.2), KIR2DS1_(NP_055327.1), KIR2DS3_(NP_036445.1), KIR2DS4_(NP_036446.3), KIR2DS5_(NP_055328.2). Sequences were aligned with MAFFT using the L-INS-I strategy and BLOSUM62 substitution matrix^37^. KIR receptor variants were identified and their occurrence estimated by cross-referencing the IPD-KIR database with NCBI’s dbSNP via the variation viewer browser^26^. The SNP accession numbers corresponding to KIR2DL3 polymorphic sites are rs528413442 (D47N), rs202032116 (L11R), rs35719984 (Q35E), R41T (rs76843526), rs78713511 (F45Y), rs145638569 (K46N), rs138897134 (H50R); accession numbers for KIR2DL1 sites are rs35509911 (R16P), rs1481853508 (G43R), rs375476159 (R50C), rs760965171 (D57H), rs765722009 (S65F), rs687485 (T70K). A table listing additional high frequency variants is shown in Table S2. The 1000Genomes_30x study was the preferred source of allele frequency data in this study^27^.

## Data availability

Crystallographic data have been deposited to the RCSB Protein Data Bank under the accession number 8TUI. All data needed to evaluate the conclusions in the paper are present in the paper or the Supplementary Materials. Request for materials will be subject to a standard material transfer agreement with the University of California.

### Supplementary Information

Supplementary Information.

## References

[1] SEER*Explorer: An interactive website for SEER cancer statistics. https://seer.cancer.gov/statistics-network/explorer/ (2023).

[2] Scott AM, Wolchok JD, Old LJ (2012). Antibody therapy of cancer. Nat Rev Cancer.

[3] Zahavi D, Weiner L (2020). Monoclonal antibodies in cancer therapy. Antibodies (Basel).

[4] Marin-Acevedo JA, Kimbrough EO, Lou Y (2021). Next generation of immune checkpoint inhibitors and beyond. Journal of Hematology & Oncology.

[5] de Miguel M, Calvo E (2020). Clinical challenges of immune checkpoint inhibitors. Cancer Cell.

[6] Galluzzi L, Humeau J, Buqué A, Zitvogel L, Kroemer G (2020). Immunostimulation with chemotherapy in the era of immune checkpoint inhibitors. Nat Rev Clin Oncol.

[7] Topalian SL, Drake CG, Pardoll DM (2015). Immune checkpoint blockade: A common denominator approach to cancer therapy. Cancer Cell.

[8] Paul S, Lal G (2017). The molecular mechanism of natural killer cells function and its importance in cancer immunotherapy. Front. Immunol..

[9] Baldwin XL, Spanheimer PM, Downs-Canner S (2023). A review of immune checkpoint blockade for the general surgeon. Journal of Surgical Research.

[10] Muntasell A (2017). Targeting NK-cell checkpoints for cancer immunotherapy. Current Opinion in Immunology.

[11] Uhrberg M (1997). Human diversity in killer cell inhibitory receptor genes. Immunity.

[12] Trowsdale J (2001). Genetic and functional relationships between MHC and NK receptor genes. Immunity.

[13] Valés-Gómez M, Reyburn HT, Mandelboim M, Strominger JL (1998). Kinetics of interaction of HLA-C ligands with natural killer cell inhibitory receptors. Immunity.

[14] Maenaka K (1999). Killer cell immunoglobulin receptors and T cell receptors bind peptide-major histocompatibility complex class I with distinct thermodynamic and kinetic properties*. Journal of Biological Chemistry.

[15] Moretta A, Locatelli F, Moretta L (2008). Human NK cells: From HLA class I-specific killer Ig-like receptors to the therapy of acute leukemias. Immunological Reviews.

[16] Foley B (2014). The biology of NK cells and their receptors affects clinical outcomes after hematopoietic cell transplantation (HCT). Immunological Reviews.

[17] Moretta, A. *et al.* P58 molecules as putative receptors for major histocompatibility complex (MHC) class I molecules in human natural killer (NK) cells. Anti-p58 antibodies reconstitute lysis of MHC class I-protected cells in NK clones displaying different specificities. *J Exp Med* **178**, 597–604 (1993). 10.1084/jem.178.2.597 PMC2191136 8340759

[18] Vahlne G (2010). In vivo tumor cell rejection induced by NK cell inhibitory receptor blockade: Maintained tolerance to normal cells even in the presence of IL-2. European Journal of Immunology.

[19] Moretta, A. *et al.* Antibodies binding to receptors Kir2dl1, -2, 3 but Not Kir2ds4 and Their Therapeutic Use. (2006).

[20] Ryser S, Estellés A, Tenorio E, Kauvar LM, Gishizky ML (2017). High affinity anti-TIM-3 and anti-KIR monoclonal antibodies cloned from healthy human individuals. PLOS ONE.

[21] Romagné F (2009). Preclinical characterization of 1–7F9, a novel human anti–KIR receptor therapeutic antibody that augments natural killer–mediated killing of tumor cells. Blood.

[22] Benson DM (2011). IPH2101, a novel anti-inhibitory KIR antibody, and lenalidomide combine to enhance the natural killer cell versus multiple myeloma effect. Blood.

[23] Carlsten M (2016). Checkpoint Inhibition of KIR2D with the monoclonal antibody IPH2101 induces contraction and hyporesponsiveness of NK cells in patients with Myeloma. Clin Cancer Res.

[24] Vey N (2018). A phase 1 study of lirilumab (antibody against killer immunoglobulin-like receptor antibody KIR2D; IPH2102) in patients with solid tumors and hematologic malignancies. Oncotarget.

[25] Zhang C, Shine M, Pyle AM, Zhang Y (2022). US-align: universal structure alignments of proteins, nucleic acids, and macromolecular complexes. Nat Methods.

[26] Robinson J, Halliwell JA, McWilliam H, Lopez R, Marsh SGE (2013). IPD—the immuno polymorphism database. Nucleic Acids Res.

[27] Byrska-Bishop M (2022). High-coverage whole-genome sequencing of the expanded 1000 Genomes Project cohort including 602 trios. Cell.

[28] Moesta AK (2008). Synergistic polymorphism at two positions distal to the ligand-binding site makes KIR2DL2 a stronger receptor for HLA-C Than KIR2DL31. The Journal of Immunology.

[29] Wong JKM, Dolcetti R, Rhee H, Simpson F, Souza-Fonseca-Guimaraes F (2023). Weaponizing natural killer cells for solid cancer immunotherapy. Trends in Cancer.

[30] Maenaka K, Juji T, Stuart DI, Jones EY (1999). Crystal structure of the human p58 killer cell inhibitory receptor (KIR2DL3) specific for HLA-Cw3-related MHC class I. Structure.

[31] Wu X (2010). Rational design of envelope identifies broadly neutralizing human monoclonal antibodies to HIV-1. Science.

[32] Otwinowski Z, Minor W (1997). Processing of X-ray diffraction data collected in oscillation mode. Methods in Enzymology.

[33] Seydoux, E. *et al.* Development of a VRC01-class germline targeting immunogen derived from anti-idiotypic antibodies. *Cell Reports* **35**, (2021). 10.1016/j.celrep.2021.109084 PMC8127986 33951425

[34] McCoy AJ (2007). Phaser crystallographic software. J Appl Cryst.

[35] Emsley P, Lohkamp B, Scott WG, Cowtan K (2010). Features and development of Coot. Acta Cryst D.

[36] Liebschner D (2019). Macromolecular structure determination using X-rays, neutrons and electrons: recent developments in Phenix. Acta Cryst D.

[37] Katoh K, Rozewicki J, Yamada KD (2019). MAFFT online service: multiple sequence alignment, interactive sequence choice and visualization. Briefings in Bioinformatics.

[38] Fan QR, Long EO, Wiley DC (2001). Crystal structure of the human natural killer cell inhibitory receptor KIR2DL1–HLA-Cw4 complex. Nat Immunol.

[39] Saunders PM (2020). The molecular basis of how buried human leukocyte antigen polymorphism modulates natural killer cell function. Proceedings of the National Academy of Sciences.

