# Supplementary material for: Structural basis for the activity and specificity of the immune checkpoint inhibitor lirilumab
Source: Sci Rep. 2024 Jan 7;14:742. doi: 10.1038/s41598-023-50262-6 (PMC10772121; doi:10.1038/s41598-023-50262-6)
Supplement: Supplementary file 1 — Supplementary Information. [file 41598_2023_50262_MOESM1_ESM.pdf]

Supplementary Materials for

Structural basis for the activity and specificity of the immune checkpoint inhibitor lirilumab

Nicholas Lorig-Roach et al.

*Corresponding author: Rebecca DuBois, rmdubois@ucsc.edu

Includes:

Figs. S1
Tables S1 and S2

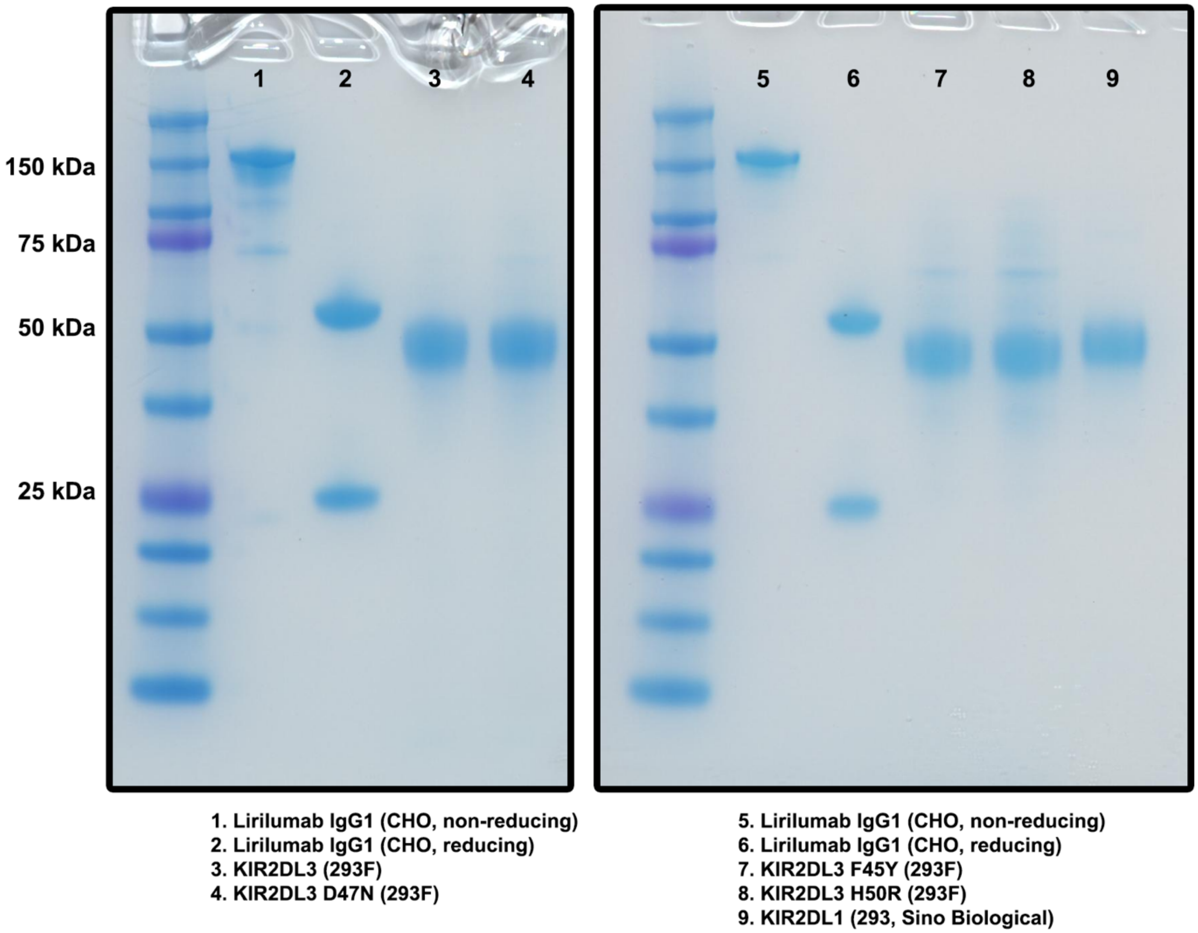

Fig. S1: SDS-PAGE gels showing lirilumab IgG1, KIR receptors used for BLI assays. Lirilumab IgG1 was purified from CHO-S cell supernatant using protein A resin. KIR receptors were purified from HEK293F supernatant using EDTA-resistant Ni-NTA followed by size exclusion chromatography.

Table S1. BLI kinetics model parameters for binding between lirilumab (IgG1) and KIR2DL1, KIR2DL3, and KIR2DL3 variants.

	Model Fit Parameters				
	K_D (nM)[a]	R^2	X^2	k_a (1/Ms)	k_d (1/s)
KIR2DL3	1.64	0.9952	0.5141	2.240E05	3.663E-04
	2.83	0.9954	0.3523	2.197E05	6.219E-04
	3.78	0.9959	0.1989	2.363E05	8.920E-04
KIR2DL1	11.5	0.9912	1.0485	8.319E04	9.537E-04
	9.6	0.9932	0.7627	1.038E05	9.946E-04
	8.8	0.9942	0.6410	1.074E05	9.475E-04
KIR2DL3 F45Y	10.25	0.9932	0.6602	1.218E05	1.249E-03
	10.8	0.9943	0.4065	1.437E05	1.554E-03
	8.5	0.9895	0.3643	1.705E05	1.450E-03
KIR2DL3 D47N[b]	1400	0.9991	0	--	--
	1900	0.9973	0.0001	--	--
KIR2DL3 H50R	7.98	0.9795	2.12	2.442E05	1.948E-03
	10.6	0.9818	1.311	2.308E05	2.437E-03
	15	0.9774	2.3512	2.116E05	3.175E-03

[a]K_D determined using Octet Data Analysis HT software's 1:1 binding model with the 'global' fit setting and using at least 4 curves for each experiment
[b]K_D determined using a steady state binding model due to poor fitting of the 1:1 model

Table S2. High frequency (>5%) inhibitory KIR2D receptor missense variants and other variants of interest

Var#[a]	KIR	Variant accession number	Genome location (GRCh38.p14)	Mutation	Minor Allele Frequency, Global (1000genomes_30x)
	KIR2DL1	rs666590	54775402	A>G = H182R	0.291
	KIR2DL1	rs147072532	54775344	G>A = D163N	0.277
	KIR2DL1	rs111799279	54775317	C>A = P154T	0.276
	KIR2DL1	rs11673144	54775198	T>C = L114P	0.266
	KIR2DL1	rs199644153	54775213	T>A = L119N	0.261
	KIR2DL1	rs597681	54778656	A>G = K216E	0.220
	KIR2DL1	rs687885	54773593	G>T = V90L	0.210
	KIR2DL1	rs574769826	54775225	A>G = N123S	0.199
Var13	KIR2DL1	rs687485	54773534	C>A = T70K	0.194
	KIR2DL1	rs2916003	54775357	G>A = G167D	0.179
	KIR2DL1	rs200746024	54775315	G>T = G153V	0.158
	KIR2DL1	rs538559888	54775318	C>G = P154R	0.155
	KIR2DL1	rs34721508	54783002	C>T = R245C	0.155
Var8	KIR2DL1	rs35509911	54773372	G>C = R16P	0.155
	KIR2DL1	rs62121640	54775329	G>A = G158R	0.151
	KIR2DL1	rs2756923	54783763	A>G = T312A	0.151
	KIR2DL1	rs2304224	54769863	Signal sequence	0.146
	KIR2DL1	rs202205602	54775305	C>T = L150F	0.065
Var10	KIR2DL1	rs375476159	54773473	C>T = R50C	No data
Var9	KIR2DL1	rs1481853508	54773452	G>A = G43R; G>C = G43R	No data
Var11	KIR2DL1	rs760965171	54773494	G>C = D57H	No data
Var12	KIR2DL1	rs765722009	54773519	C>T = S65F	No data
	KIR2DL3	rs3810343	54738571	Signal sequence	0.361
Var2	KIR2DL2/3	rs35719984	54742075	C>G = Q35E	0.340
	KIR2DL3	rs1049267	54752446	G>A = R297H; G>C = R297P	0.236 (A) 0.160 (C)
	KIR2DL2/3	rs200686594	54743929	C>T = R148C	0.194
	KIR2DL2/3	rs150145497	54744086	C>T = T200I	0.149
Var4	KIR2DL2/3	rs78713511	54742106	T>A = F45Y	0.135
	KIR2DL2/3	rs1555897648	54742019	C>G = P16R	0.134
	KIR2DL2/3	rs201504521	54743909	G>C = R141T	0.120
	KIR2DL3	rs35861855	54747356	C>T = P208L	0.115
	KIR2DL2/3	rs200359536	54744000	C>A = H171N	0.096
	KIR2DL2/3	rs200426472	54743878	C>T = R131W	0.096
	KIR2DL3	rs2365227	54752222	T>C = V255A	0.085
	KIR2DL3	rs4020198	54752265	G>C = R279S	0.079
Var7	KIR2DL2/3	rs138897134	54742121	A>G = H50R	0.057
Var1	KIR2DL2/3	rs202032116	54742004	T>G = L11R	0.053
Var3	KIR2DL2/3	rs76843526	54742094	G>C = R41T	0.006
Var6	KIR2DL2/3	rs528413442	54742111	G>A = D47N	0.001
Var5	KIR2DL2/3	rs145638569	54742110	G>C = K46N	0.0002

[a]Var#s correspond to those listed in Fig. 4.
